# Socioeconomic differences in the association between maternal age and maternal obesity: a register-based study of 707,728 women in Finland

**DOI:** 10.1177/14034948221088003

**Published:** 2022-05-20

**Authors:** Zahra Roustaei, Sari Räisänen, Mika Gissler, Seppo Heinonen

**Affiliations:** 1 Department of Health Sciences, University of Helsinki, Helsinki, Finland; 2 School of Health Care and Social Services, Tampere University of Applied Sciences, Tampere, Finland; 3 Department of Knowledge Brokers, Finnish Institute for Health and Welfare (THL), Helsinki, Finland; 4 Academic Primary Health Care Centre, Region Stockholm, Stockholm, Sweden; 5 Department of Molecular Medicine and Surgery, Karolinska Institute, Stockholm, Sweden; 6 Department of Obstetrics and Gynecology, University of Helsinki and Helsinki University Hospital, Helsinki, Finland

**Keywords:** Maternal age, maternal obesity, socioeconomic status, register-based study

## Abstract

**Aims:**

To examine the association between maternal age and maternal obesity across socioeconomic groups and to determine whether socioeconomic status modifies the association between maternal age and maternal obesity with a view to informing public health policies.

**Methods:**

Data for this register-based study were sourced from the Finnish Medical Birth Register and Statistics Finland, using the information of 707,728 women who gave birth in Finland from 2004 to 2015. We used multivariable regression models to assess the association between maternal age and maternal obesity across socioeconomic groups. We further assessed interactions on both multiplicative and additive scales.

**Results:**

Across all socioeconomic groups, the adjusted odds ratio for the association between maternal age and maternal obesity increased, peaking for women 35 years or older. Using women below 20 years of age in the category of upper-level employees as a single reference group, in the category of upper-level employees, the adjusted odds ratio and 95% confidence intervals among women 35 years or older was 1.92 (1.39–2.64) for maternal obesity. Equally, the adjusted odds ratio and 95% confidence intervals in the category of long-term unemployed was 4.35 (3.16–5.98). Synergistic interactions on both multiplicative and additive scales were found across age and socioeconomic groups.

**Conclusions:**

**The association between maternal age and maternal obesity was strongest among women 35 years or older with lower socioeconomic status. Population-level interventions that address maternal risk factors from teenage years are needed alongside individual-level interventions that target high-risk mothers in areas of low socioeconomic status and maternal obesity.**

## Background

Pregnancy outcomes largely explain health and behavioural characteristics of women before and during pregnancy, and poorer health and pregnancy outcomes related to obesity are of concern, as obesity represents a rapidly growing maternal risk factor [1, 2]. For example, in Finland the proportion of women with obesity has risen from 7.5% in 1990 to 17.0% in 2020 [2, 3]. This is particularly concerning because obesity is an important risk factor for infertility, and women with obesity who become pregnant are at increased risk of short and long-term adverse consequences as well as adverse offspring outcomes [4].

Women with low socioeconomic status have a higher risk of overweight and obesity than women with high socioeconomic status, even after controlling for individual-level risk factors [5, 6]. Therefore, the prevalence of maternal risk factors partly explains the excess risk of obesity associated with low socioeconomic status. Pathways that give rise to this increased risk of overweight and obesity are not well understood but might include extremes of unhealthy behaviour, reduced access to healthcare services, and interactions between biological and social processes [6–8]. For the latter mechanism, Geronimus et al. suggested that the cumulative and interactive impact of socioeconomic stressors associated with low socioeconomic status over a life span can contribute to poorer health outcomes [9]. This disproportionate rate of poor health outcomes, such as adverse pregnancy outcomes by maternal age has been mainly studied in the United States, while rapidly increasing maternal risk factors (such as obesity) in welfare societies have been studied less [10]. Studies have indicated that part of the association between low socioeconomic status and maternal obesity could be explained by maternal age [5, 6], but it is unknown whether the increased risk of obesity among older women is due to the biological response or an increased prevalence of other risk factors associated with low socioeconomic status. Study of the interaction between maternal age and socioeconomic status might improve our understanding of the excess risk of maternal obesity associated with low socioeconomic status.

This study aimed to assess the association between maternal age and maternal obesity across socioeconomic groups to determine how maternal occupation as a proxy for socioeconomic status affects the association between age and maternal obesity, and to inform public health policies by identifying vulnerable women who could benefit most from interventions. We hypothesised that the risk of maternal obesity increases disproportionately with increasing age across socioeconomic groups, and this disproportionate risk is greater when women have advanced maternal age and low socioeconomic status together.

## Methods

We undertook a register-based study of 707,728 women who gave birth in Finland from 2004 to 2015. Our analyses were based on the data from the Finnish Medical Birth Register (MBR) and Statistics Finland. In Finland, maternity care is provided free of charge in public facilities, and all women have access to healthcare services.

The MBR is a population-based registry and contains individual-level information on sociodemographic background, previous and present pregnancies, and deliveries of all women who have given birth in Finland [11]. The MBR was established in 1987 and is maintained by the Finnish Institute for Health and Welfare (THL). All birth hospitals transmit data to THL for pregnancies with at least 22 weeks of gestation or a birth weight of 500 g or more. Information on socioeconomic status was gathered from Statistics Finland. We linked the information from the MBR and Statistics Finland by using unique national identification numbers. The statistical authorities removed identifiers after completing the data linkages. Approval for using the data for this study was obtained from THL (permission number THL/876/5.05.00/2017).

From the MBR, we calculated body mass index (BMI) from self-reported information about height and prepregnancy weight, recorded and checked at the first antenatal visit between 8 and 12 weeks of gestation by midwives, to classify the weight status of women. These data have been collected in the MBR since 2004. We calculated BMI as the weight in kilograms divided by height in meters squared, and categorised women as having maternal obesity or not using BMI 30 (kg/m^2^) as a cutoff point, according to the criteria recommended by the World Health Organization (1995) [12]. BMI was missing for 5.1% (*n*=35,986) of women.

We categorised variable maternal age according to most of the literature on maternal health disparities as 19 or less, 20–24, 25–29, 30–34 and 35 or greater. We considered women below 20 years of age the reference group because their risk of maternal obesity was the lowest. Advanced maternal age was defined as maternal age 35 years or older.

Socioeconomic status was determined based on maternal occupation and was obtained at the first pregnancy interview from the MBR and on year-end data on occupational class from the register of Statistics Finland. We used the socioeconomic classification of Statistics Finland as a general indicator of the social and economic environment of women [13]. We focused on the following categories: self-employed persons; upper-level employees (administrative, managerial, professional and related occupations); lower-level employees (administrative and clerical occupations); manual workers; students; long-term unemployed; others. Others included those not elsewhere classified and socioeconomic status unknown. We considered upper-level employees as a reference group as their risk of obesity was the lowest. Missing information on socioeconomic status comprised 13.5% (*n*=95,510) of all births. We created a separate category for women with missing information on socioeconomic status in the analyses.

The covariates included in the analyses were identified based on a directed acyclic graph (DAG) (Supplemental Figure 1). Information on smoking was self-reported and was divided into non-smokers, stopped smoking during the first trimester, continued to smoke after the first trimester and missing information. We categorised parity according to the self-reported total number of live births into two groups: no previous birth (nulliparity) and one or more previous births (multiparity). Marital status was classified as married/cohabiting and single.

The data analyses were performed using SPSS version 27.0 and Stata version 16.0. We analysed the data at the significance level of 0.05. We used Pearson’s chi-square test to assess whether there was an association between a categorical variable and maternal age or to investigate whether there was a statistically significant difference in the distribution of a categorical variable and categories of maternal age.

We explored the associations between maternal age and maternal obesity using multivariable logistic models. We ran separate models for each socioeconomic stratum as a covariate that reflects maternal education, occupation and income. We further included parity in the analysis. Parity is a correlate of maternal obesity as a higher number of births are associated with a higher risk of maternal obesity, and multiple cycles of pregnancy are more likely to occur with increasing maternal age. In addition, the number of children prior to the pregnancy may affect the timing of the subsequent pregnancy.

Furthermore, we performed several multivariable analyses to estimate the associations between maternal age and socioeconomic status with maternal obesity. We first evaluated the unadjusted model. The first model adjusted for parity. The final model adjusted for socioeconomic status, parity, smoking and marital status.

We evaluated the effect modification on both additive and multiplicative scales, as recommended [14]. We assessed the presence of interaction on the multiplicative scale by fitting an interaction term between maternal age and socioeconomic status. To illustrate the interaction effect, we calculated adjusted odds ratios (aORs) for each stratum with a single reference category. In addition, we provided interactions on the additive scale because departures from the additive model have implications for public health interventions [14]. For that, we calculated the relative excess risk due to interaction (RERI) to identify subgroups of women who are at increased cumulative risk of maternal obesity, using Stata codes for additive interaction for categorical exposures provided by VanderWeele and Knol [14].

## Results

Of 707,728 women giving birth in Finland from 2004 to 2015, most (59.2%, 418,942) had normal BMI, 20.6% (145,566) were overweight, and 11.5% (81,133) were women with obesity. The proportion of women with obesity increased from 8.6% (4941) in 2004 to 12.9% (7197) in 2015 (Supplemental Table I).

Characteristics of women by maternal age are presented in Table I. The greatest proportion of maternal overweight or obesity was among women 35 years or older, and the lowest proportion was experienced by teenage mothers (Table I, Supplemental Figure 2). Mothers 35 years or older had a greater proportion of multiparity (Table I). If we stratified maternal obesity by socioeconomic groups, long-term unemployed women and women in manual occupations had higher proportions of smokers, being single in pregnancy, and women with obesity than other socioeconomic groups (Supplemental Table II).

**Table I.** Characteristics of the study population by maternal age, from 2004 to 2015 in Finland.

	Maternal age (years)				
Total (*n*, %)^a^ 707,728 (100)	≤19 16,214 (2.3%) ^c^	20–24 108,689 (15.4%)	25–29 222,399 (31.4%)	30–34 225,055 (31.8%)	≥35 135,371 (19.1%)
Characteristic^b^					
Socioeconomic status (*n*, %)					
Upper-level employees	933 (5.8)	6364 (5.9)	11,995 (5.4)	18,461 (8.2)	21,621 (16.0)
Self-employed persons	1206 (7.4)	7441 (6.8)	7860 (3.5)	3950 (1.8)	4834 (3.6)
Lower-level employees	2471 (15.2)	16,426 (15.1)	37,186 (16.7)	49,416 (22.0)	38,432 (28.4)
Manual workers	5354 (33.0)	29,379 (27.0)	45,844 (20.6)	38,006 (16.9)	20,611 (15.2)
Students	712 (4.4)	20,338 (18.7)	66,303 (29.8)	61,342 (27.3)	16,226 (12.0)
Long-term unemployed	2086 (12.9)	9363 (8.6)	15,801 (7.1)	16,317 (7.3)	10,863 (8.0)
Others	1582 (9.8)	7099 (6.5)	12,128 (5.5)	11,154 (5.0)	6500 (4.8)
No information	1870 (11.5)	12,279 (11.3)	25,282 (11.4)	26,409 (11.7)	16,284 (12.0)
Parity (*n*, %)					
Nulliparous	12,085 (74.5)	52,436 (48.2)	83,720 (37.6)	57,657 (25.6)	21,312 (15.7)
Multiparous No information	4117 (25.4) 12 (0.1)	56,159 (51.7) 94 (0.1)	138,497 (62.3) 182 (0.1)	167,269 (74.3) 129 (0.1)	113,987 (84.2) 72 (0.1)
Body mass index categories (*n*, %)					
Underweight (<18.5 kg/m^2^)	1578 (9.7)	6947 (6.4)	7989 (3.6)	5612 (2.5)	2498 (1.8)
Normal weight (18.5–24.9 kg/m^2^)	10,021 (61.8)	64,184 (59.1)	135,193 (60.8)	13,535 (60.1)	74,193 (54.8)
Overweight (25.0–29.9 kg/m^2^)	2509 (15.5)	20,356 (18.7)	43,676 (19.6)	46,888 (20.8)	32,137 (23.7)
Obese (⩾30.0 kg/m^2^)	1103 (6.8)	11,936 (11.0)	24,505 (11.0)	25,289 (11.2)	18,300 (13.5)
No information	1003 (6.2)	5266 (4.8)	11,036 (5.0)	11,915 (5.3)	8243 (6.1)
Smoking status (*n*, %)					
Non-smokers	7861 (48.5)	74,136 (68.2)	184,649 (83.0)	197,734 (87.9)	119,224 (88.1)
Quit during the first trimester	2073 (12.8)	10,305 (9.5)	12,479 (5.6)	8286 (3.7)	3771 (2.8)
Smokers	5811 (35.8)	21,444 (19.7)	19,951 (9.0)	13,578 (6.0)	8742 (6.5)
No information	469 (2.9)	2804 (2.6)	5320 (2.4)	5457 (2.4)	3634 (2.7)
Marital status (*n*, %)					
Married or registered partnership	2664 (16.4)	40,534 (37.3)	128,361 (57.7)	149,897 (66.6)	88,963 (65.7)
Single	13,545 (83.5)	68,132 (62.7)	94,007 (42.3)	75,119 (33.4)	46,389 (34.3)
No information	5 (0.0)	23 (0.0)	31 (0.0)	39 (0.0)	19 (0.0)

a Row percentage.

b Column percentages.

c Data presented as number and percentage.

All maternal age differences were statistically significant at *P*<0.001.

In socioeconomic status, others included not elsewhere classified and socioeconomic status unknown, in smoking status, smokers refer to those who did not quit smoking during the first trimester.

Associations between maternal age and maternal obesity were evaluated in each socioeconomic status stratum (Table II). Relative to women below 20 years of age, there were marked increases in aORs for maternal obesity across maternal age groups in all socioeconomic groups (Table II). Adjusting for parity attenuated the association between maternal age and maternal obesity across all socioeconomic groups (crude results Supplemental Table III).

**Table II.** Multivariable logistic regression models of the association between maternal age and maternal obesity, stratified by socioeconomic groups.

	≤19	20–24	25–29	30–34	≥35
	Adjusted OR	Adjusted OR (95% CI)	Adjusted OR (95% CI)	Adjusted OR (95% CI)	Adjusted OR (95% CI)
Socioeconomic groups					
Upper-level employees	1.00 (ref)	1.63 (1.18–2.27)	1.57 (1.14–2.17)	1.27 (0.92–1.76)	1.79 (1.30–2.47)
Self-employed persons	1.00 (ref)	1.28 (1.03–1.59)	1.29 (1.04–1.61)	1.45 (1.15–1.83)	1.64 (1.31–2.06)
Lower-level employees	1.00 (ref)	1.69 (1.43–2.01)	1.52 (1.29–1.80)	1.50 (1.27–1.77)	1.92 (1.62–2.27)
Manual workers	1.00 (ref)	1.74 (1.55–1.94)	1.70 (1.52–1.90)	1.81 (1.62–2.03)	2.40 (2.14–2.69)
Students	1.00 (ref)	2.05 (1.43–2.93)	2.12 (1.48–3.02)	2.08 (1.45–2.97)	2.42 (1.69–3.46)
Long-term unemployed	1.00 (ref)	1.64 (1.37–1.95)	1.72 (1.45–2.05)	1.96 (1.64–2.33)	2.30 (1.93–2.74)
Others	1.00 (ref)	1.52 (1.23–1.87)	1.54 (1.25–1.88)	1.67 (1.36–2.05)	2.17 (1.76–2.68)
No information	1.00 (ref)	1.53 (1.43–1.63)	1.49 (1.40–1.58)	1.47 (1.38–1.57)	1.79 (1.68–1.91)

OR: odds ratio; CI: confidence interval.

Women in the category of ⩽19 years of age for each socioeconomic group were used as the reference group.

In socioeconomic status, others included not elsewhere classified and socioeconomic status unknown.

Analyses were adjusted for parity.

Table III presents the odds ratios (ORs) and aORs with 95% confidence intervals (CIs) for the association between maternal age, socioeconomic status, and other covariates and maternal obesity. In crude analyses, the highest risk of obesity during pregnancy (OR >2) was observed among women 35 years or older and long-term unemployed women. Adjusting for parity in model 1 showed that multiparity explained part of the association between maternal age and obesity, especially for women 35 years or older. In model 2, aORs for the associations between maternal age and maternal obesity were stronger than in model 1. The association between socioeconomic status and maternal obesity was statistically significant for all socioeconomic groups in comparison with upper-level employees. The association was strongest for long-term unemployed women (aOR 2.19, 95% CI 2.11, 2.28). Supplemental Figure 3 shows the probability of maternal obesity predicted by maternal age and socioeconomic status based on logistic regression coefficients from the fully adjusted model (Supplemental Figure 3).

**Table III.** Associations between maternal age, socioeconomic status, and maternal obesity.

Outcome	Crude	Model 1	Model 2
	Adjusted OR (95% CI)	Adjusted OR (95% CI)	Adjusted OR (95% CI)
Maternal age (years)			
≤19	1.00 (ref)	1.00 (ref)	1.00 (ref)
20–24	1.66 (1.56–1.77)	1.53 (1.43–1.63)	1.73 (1.62–1.85)
25–29	1.67 (1.57–1.78)	1.49 (1.39–1.58)	1.84 (1.72–1.96)
30–34	1.72 (1.61–1.83)	1.47 (1.38–1.57)	1.90 (1.78–2.02)
≥35	2.15 (2.01–2.29)	1.79 (1.68–1.91)	2.37 (2.22–2.54)
Parity			
Nulliparous	1.00 (ref)	1.00 (ref)	1.00 (ref)
Multiparous	1.41 (1.39–1.44)	1.36 (1.34–1.38)	1.31 (1.29–1.33)
Socioeconomic status			
Upper-level employees	1.00 (ref)		1.00 (ref)
Self-employed persons	1.71 (1.62–1.79)		1.79 (1.70–1.88)
Lower-level employees	1.54 (1.48–1.59)		1.54 (1.49–1.60)
Manual workers	1.92 (1.86–1.99)		1.98 (1.91–2.05)
Students	1.47 (1.42–1.52)		1.56 (1.51–1.62)
Long-term unemployed	2.22 (2.14–2.31)		2.19 (2.11–2.28)
Others	1.94 (1.86–2.03)		1.94 (1.86–2.03)
No information	1.58 (1.52–1.64)		1.62 (1.56–1.69)
Smoking status			
Non-smokers	1.00 (ref)		1.00 (ref)
Smokers	1.50 (1.47–1.54)		1.46 (1.43–1.49)
Marital status (*n*, %)			
Single	1.00 (ref)		1.00 (ref)
Married or registered partnership	1.04 (1.02–1.05)		1.04 (1.02–1.06)
Intercept		0.072	0.033

OR: odds ratio; CI: confidence interval.

Model 1: adjusted by parity.

Model 2: adjusted by parity with the addition of socioeconomic status, smoking, and marital status.

In socioeconomic status, others included not elsewhere classified and socioeconomic status unknown, in smoking status, smokers refer to those who did not quit smoking during the first trimester.

When all groups were compared with a single combined category of women below 20 years of age and upper-level employees as the reference group, the risk of maternal obesity across maternal age groups varied by socioeconomic status, as presented in Table IV. Among women in the category of upper-level employees, the aOR for women in the category of 35 years or older was 1.92 (95% CI 1.39, 2.64) for maternal obesity. In contrast, women 35 years or older who were unemployed had an aOR of 4.30 (95% CI 3.13, 5.92) for maternal obesity. As shown in Table IV, there was a positive interaction with aOR greater than 1 on the multiplicative scale between maternal age and socioeconomic status for maternal obesity (*P*_interaction_<0.001). Interaction analyses on the additive scale showed that the joint effect of maternal age and socioeconomic status on maternal obesity exceeded the sum of their individual effects in subgroups of women with RERI greater than 0 (Table V and Supplemental Table IV).

**Table IV.** Multivariable logistic regression models of the association between maternal age, socioeconomic status and maternal obesity.

Outcome	≤19	20–24	25–29	30–34	≥35	*P*_interaction_
Maternal obesity	Adjusted OR (95% CI)	Adjusted OR (95% CI)	Adjusted OR (95% CI)	Adjusted OR (95% CI)	Adjusted OR (95% CI)	Adjusted OR (95% CI)
Upper-level employees	1.00 (ref)	1.70 (1.23–2.37)	1.68 (1.22–2.32)	1.38 (1.00–1.91)	1.92 (1.39–2.64)	<0.001 1.014 (1.013–1.015)
Self-employed persons	1.88 (1.29–2.73)	2.58 (1.87–3.57)	2.65 (1.92–3.66)	3.00 (2.16–4.17)	3.32 (2.40–4.60)	
Lower-level employees	1.36 (0.95–1.94)	2.51 (1.82–3.45)	2.37 (1.72–3.25)	2.32 (1.69–3.19)	2.99 (2.18–4.10)	
Manual workers	1.48 (1.06–2.07)	2.80 (2.04–3.84)	2.91 (2.12–3.99)	3.15 (2.29–4.32)	4.15 (3.03–5.70)	
Students	1.06 (0.66–1.70)	2.23 (1.62–3.06)	2.44 (1.78–3.35)	2.45 (1.78–3.35)	2.85 (2.07–3.92)	
Long-term unemployed	1.57 (1.10–2.24)	2.85 (2.07–3.92)	3.20 (2.33–4.39)	3.75 (2.73–5.15)	4.35 (3.16–5.98)	
Others	1.56 (1.08–2.26)	2.58 (1.87–3.56)	2.82 (2.05–3.89)	3.15 (2.29–4.33)	4.14 (3.00–5.71)	
No information	1.72 (1.20–2.47)	2.24 (1.63–3.09)	2.50 (1.82–3.43)	2.55 (1.85–3.50)	3.14 (2.28–4.31)	

OR: odds ratio; CI; confidence interval.

Data presented as adjusted OR (95% CI) per maternal age and socioeconomic status groups with women in the category of ⩽19 years of age and upper-level employees used as the sole reference group (ref) for maternal obesity.

*P*_interaction_ indicates the results of statistical interaction between maternal age and socioeconomic status.

Others included not elsewhere classified and socioeconomic status unknown.

The adjustment has been made for the relationship between both exposures and maternal obesity.

Models were adjusted for parity, smoking, and marital status.

**Table V.** The relative excess risk due to interaction (RERI) and 95% confidence interval for maternal obesity.

Outcome	≤19	20–24	25–29	30–34	≥35
Maternal obesity	Estimates (95% CI)	Estimates (95% CI)	Estimates (95% CI)	Estimates (95% CI)	Estimates (95% CI)
Upper-level employees	(ref)	(ref)	(ref)	(ref)	(ref)
Self-employed persons	(ref)	0.05 (-0.50–0.61)	0.08 (–0.45–0.63)	0.84 (0.30–1.38)	0.72 (0.19–1.25)
Lower-level employees	(ref)	0.48 (0.13–0.83)	0.35 (0.01–0.69)	0.59 (0.30–0.88)	0.79 (0.51–1.07)
Manual workers	(ref)	0.65 (0.37–0.92)	0.69 (0.46–0.93)	1.19 (0.97–1.42)	1.75 (1.39–2.11)
Students	(ref)	0.50 (0.05–0.95)	0.73 (0.33–1.13)	1.14 (0.76–1.5)	1.03 (0.61–1.45)
Long-term unemployed	(ref)	0.60 (0.22–0.98)	0.85 (0.52–1.18)	1.70 (1.29–2.11)	1.78 (1.32–2.24)

CI: confidence interval.

Women in the ‘upper-level’ category and maternal age <19 used as the reference groups (ref) for maternal obesity in each four ‘exposure’ groups of women; for example, upper-level employees and manual occupations with women age ⩽19 years and women in the category of 20 to 24 years (RERI=0.65).

The adjustment has been made for the relationship between both exposures and maternal obesity.

All analyses were adjusted for variables parity, smoking, and marital status.

Interaction on the additive scale was present if the 95% CIs of RERI did not include 0.

## Discussion

Our results show that the category of women aged 35 years or more has the strongest association with maternal obesity across all socioeconomic groups. The association between advanced maternal age and maternal obesity became stronger among women with lower socioeconomic status. Women in manual occupations and unemployed women aged 35 years or more had disproportionately higher aORs for maternal obesity compared with women with higher socioeconomic status below 20 years of age (Table IV). This socioeconomic difference across age groups was not accounted for by differences in parity, smoking or marital status. Moreover, we found maternal age and socioeconomic status operated in super-multiplicative and super-additive ways.

Our findings are in line with studies that have used education as a proxy for socioeconomic status and shown that socioeconomic difference in maternal obesity exists in early adulthood and widens until middle-age in women [15]. Although in our population, women with lower socioeconomic status had a higher proportion of co-occurring maternal risk factors (differential exposure), this disproportionate maternal age-associated risk of maternal obesity among women with lower socioeconomic status was not accounted for by differences in the prevalence of maternal risk factors. In our population, women who had postponed pregnancy were increasingly more vulnerable to the deleterious effect of low socioeconomic status (differential effect), supporting the weathering hypothesis [5]. According to this hypothesis, maternal age represents a period during which chronic exposure to social and economic stressors accumulates to a sufficient extent to trigger poor health outcomes. This triggering point of poor health outcomes could be shorter among women of lower socioeconomic status due to a high level of chronic exposure to obesity risk factors from the sensitive teenage period, and because women with lower socioeconomic status are more vulnerable to these risk factors than women in high socioeconomic groups [16–18].

Among upper-level employees, the association between maternal age and maternal obesity across all age groups was weaker than that of other socioeconomic groups. In Finland, upper-level employees are usually highly educated and are more likely to have a higher income and live in a high socioeconomic area. This suggests the possibility that personal, social and economic resources are distributed differently across socioeconomic groups. Another possibility is that educated women comply better with diet and lifestyle recommendations before pregnancy, and are consequently more likely to have normal prepregnancy BMI and are less likely to have high postpartum weight retention [19, 20].

Consistent with other studies, the maternal age–obesity association was attenuated after the inclusion of parity because of the direct effect of biological changes during pregnancy on body weight [7, 20, 21]. In the Finnish population, the socioeconomic difference in completed fertility is small, but Finnish women with high socioeconomic status are more likely to postpone pregnancy and have short birth intervals which are positively associated with maternal obesity [22]. In contrast, women with low socioeconomic status may experience having an early first birth [15], a longer period of exposure to weight-related risk factors associated with motherhood, and consequently experience a steeper weight trajectory [20, 23, 24]. Nevertheless, our results indicated that the postponement of pregnancy among low socioeconomic groups, after controlling for the time-varying effect of parity in each maternal age and socioeconomic group, was not associated with a similar risk of maternal obesity in upper-level employees.

Another potential explanation for the apparent increased risk of maternal obesity among older women could be the potential for reverse causation. It is likely that obesity-related infertility results in higher maternal age. However, there was little evidence for reverse causation in this study (Supplemental Table V).

We used Finnish MBR data in this study, which have been recognised to be valid, because of the coverage of more than 99.9% of all births in Finland and the quality of the data [25]. This study had sufficient statistical power to test the hypotheses of interaction and to identify high-risk groups because of the large sample size. The MBR data linked with the information from Statistics Finland have been used in previous studies to study maternal health inequality, because the data contain reliable information on socioeconomic status and health across reproductive years, free of attrition and non-response bias, for a large number of women [26]. In the current study we had no information on maternal education and income. However, occupation is considered to be a good measure of socioeconomic status in this study, as the occupation is interrelated with education and income in Finland [27].

The results of this study should be interpreted by bearing several caveats in mind. In this study, misreporting of weight and height could result in misclassification of women into BMI categories. If underreporting of weight and overreporting of height occurred in our study, the proportion of maternal obesity in our study could be underestimated, in line with the misreporting phenomenon [28]. However, self-reported height and weight were checked at the first prenatal visits to assess whether the information was consistent with the real measurements. In addition, self-reported height and weight have been shown to be accurate enough in the Finnish population for large studies [28]. Although we have been able to control for important covariates in our analyses, the Finnish MBR does not record weight changes during pregnancy and lifestyle-related risk factors associated with maternal obesity.

Our findings highlight the potential importance of interventions among teenage women with lower socioeconomic status, because their socioeconomic differences with upper-level employees are not yet wide. In addition, lifestyle interventions should not be limited to the short duration of pregnancy, because a meta-analysis of 36 studies found that interventions during pregnancy on average gave a reduction of only 0.7 kg in gestational weight gain, with no significant impact on pregnancy outcomes [29]. Thus, interventions need to be implemented at the population level from the teenage years to promote health in general, while acknowledging the socioeconomic environments of women during the reproductive years. Moreover, careful and timely obstetric interventions among high-risk mothers are important, because in high-income countries advanced maternal age, obesity and high-risk pregnancies are becoming increasingly common [1]. In this study, by using the interaction on the additive scale we have been able to identify subgroups of women who are most likely to benefit from the targeted interventions [30]. For example, unemployed women aged 35 years or older, who are most vulnerable to maternal obesity, may have the largest reduction in obesity risk from targeted interventions for one or more exposures.

## Conclusions

Maternal obesity was most common among women 35 years or older across all socioeconomic groups. The association between maternal age and maternal obesity was stronger in lower socioeconomic groups. It is important that policies at the population level address maternal risk factors related to obesity especially among low socioeconomic groups from a sensitive period of the teenage years. Simultaneously, at the individual level interventions among high-risk women could reap the largest reduction in maternal obesity at the population level in Finland. This reduction in the risk of maternal obesity would not only come about because of the higher prevalence of maternal risk factors pertaining to women with low socioeconomic status at different age groups, but because women with lower socioeconomic status seem to be more vulnerable to have maternal obesity with increasing maternal age.

## Supplemental Material

sj-docx-1-sjp-10.1177_14034948221088003 – Supplemental material for Socioeconomic differences in the association between maternal age and maternal obesity: a register-based study of 707,728 women in Finland Click here for additional data file. Supplemental material, sj-docx-1-sjp-10.1177_14034948221088003 for Socioeconomic differences in the association between maternal age and maternal obesity: a register-based study of 707,728 women in Finland by Zahra Roustaei, Sari Räisänen, Mika Gissler and Seppo Heinonen in Scandinavian Journal of Public Health

sj-docx-2-sjp-10.1177_14034948221088003 – Supplemental material for Socioeconomic differences in the association between maternal age and maternal obesity: a register-based study of 707,728 women in Finland Click here for additional data file. Supplemental material, sj-docx-2-sjp-10.1177_14034948221088003 for Socioeconomic differences in the association between maternal age and maternal obesity: a register-based study of 707,728 women in Finland by Zahra Roustaei, Sari Räisänen, Mika Gissler and Seppo Heinonen in Scandinavian Journal of Public Health
