# Supplementary material for: Socioeconomic differences in the association between maternal age and maternal obesity: a register-based study of 707,728 women in Finland
Source: Scand J Public Health. 2022 May 20;51(6):963–71. doi: 10.1177/14034948221088003 (PMC10350730; doi:10.1177/14034948221088003)
Supplement: sj-docx-2-sjp-10.1177_14034948221088003 – Supplemental material for Socioeconomic differences in the association between maternal age and maternal obesity: a register-based study of 707,728 women in Finland [file sj-docx-2-sjp-10.1177_14034948221088003.docx]

**Socioeconomic differences in the association between maternal age and maternal obesity: a register-based study of 707 728 women in Finland from 2004 to 2015**

Zahra Roustaei ^1^, Sari Räisänen ^2^, Mika Gissler ^3,4, 5^, Seppo Heinonen ^6^

^1^Department of Health Sciences, University of Helsinki, 00014 Helsinki, Finland.

^2^School of Health Care and Social Services, Tampere University of Applied Sciences, Tampere, Finland.

^3^Information Services Department, Finnish Institute for Health and Welfare (THL), Helsinki, Finland.

^4^ Academic Primary Health Care Centre, Region Stockholm, Stockholm, Sweden

^5^Department of Molecular Medicine and Surgery, Karolinska Institute, Stockholm, Sweden.

^6^Department of Obstetrics and Gynecology, University of Helsinki and Helsinki University Hospital, Helsinki, Finland.

**Contact info:** Zahra Roustaei [zahraa.roustaei@gmail.com](mailto:zahraa.roustaei@gmail.com)

**Supporting Information**

Figure S1: Direct acyclic graph for the association between maternal age (the primary exposure) and socioeconomic status (the secondary exposure) and maternal obesity.


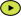
 exposure,
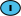
 outcome,
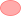
 ancestor of exposure and outcome,
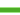
 causal path,
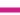
 biasing path.

Table S1: The proportion of women with maternal obesity in each year from 2004 to 2015

| Total | 2004-2015  707 728 | 2004 57 759 | 2005 57 819 | 2006 59 051 | 2007 58 933 | 2008  59 801 | 2009  60 790 | 2010  61 372 | 2011  60 257 | 2012  59 857 | 2013  58 525 | 2014  57 805 | 2015  55 759 |
| --- | --- | --- | --- | --- | --- | --- | --- | --- | --- | --- | --- | --- | --- |
| Maternal obesity | 81 133 (11.5) ^a^ | 4 941  (8.6) | 5 326 (9.2) | 6 299 (10.7) | 6 398  (10.9) | 6 551  (11.0) | 7 001 (11.5) | 7 217 (11.8) | 7 468 (12.4) | 7 598 (12.7) | 7 712 (13.2) | 7 425 (12.8) | 7 197 (12.9) |

^a^ Data presented as number and percentage.


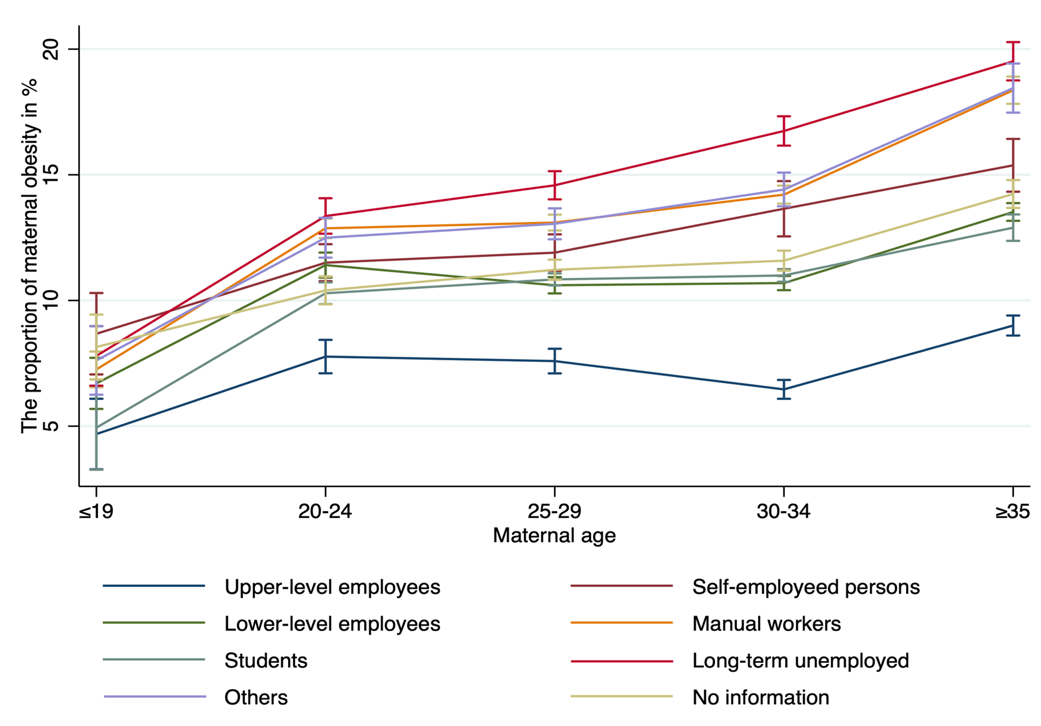


Figure S2 The proportion of maternal obesity by maternal age and socioeconomic status from 2004 to 2015 in Finland. Error bars indicate 95% CIs.

Table S2: Characteristics of the study population by socioeconomic status

| Characteristic |  | | | | | | | |  |
| --- | --- | --- | --- | --- | --- | --- | --- | --- | --- |
|  | Self-employed persons | Upper-level employees | Lower-level employees | Manual workers | Students | Long-term unemployed | Others | No information | |
| *Total (n, %) ^a^* | 25 291 (3.6) ^c^ | 59 374 (8.4) | 143 931 (20.3) | 139 194 (19.7) | 164 921 (23.3) | 54 430 (7.7) | 38 463 (5.4) | 82 124 (11.6) | |
| 707 728 |  |  |  |  |  |  |  |  | |
| *Characteristic ^b^* |  |  |  |  |  |  |  |  | |
| *Maternal age* |  |  |  |  |  |  |  |  | |
| <19 | 1 206 (4.8) | 933 (1.6) | 2 471 (1.7) | 5 354 (3.8) | 712 (0.4) | 2 086 (3.8) | 1 582 (4.1) | 1 870 (2.3) | |
| 20-24 | 7 441 (29.4) | 6 364 (10.7) | 16 426 (11.4) | 29 379 (21.1) | 20 338 (12.3) | 9 363 (17.2) | 7 099 (18.5) | 12 279 (15.0) | |
| 25-29 | 7 860 (31.1) | 11 995 (20.2) | 37 186 (25.8) | 45 844 (32.9) | 66 303 (40.2) | 15 801 (29.0) | 12 128 (31.5) | 25 282 (30.8) | |
| 30-34 | 3 950 (15.6) | 18 461 (31.1) | 49 416 (34.3) | 38 006 (27.3) | 61 342 (37.2) | 16 317 (30.0) | 11 154 (29.0) | 26 409 (32.2) | |
| ≥35 | 4 834 (19.1) | 21 621 (36.4) | 38 432 (26.7) | 20 611 (14.8) | 16 226 (9.8) | 10 863 (20.0) | 6 500 (16.9) | 16 284 (19.8) | |
| *Marital status (n, %)* |  |  |  |  |  |  |  |  | |
| Married or registered partnership | 14 240 (56.3) | 40 890 (68.9) | 85 341 (59.3) | 70 713 (50.8) | 99 641 (60.4) | 29 254 (53.7) | 20 753 (54.0) | 49 587 (60.4) | |
| Single | 11 050 (43.7) | 18 479 (31.1) | 58 580 (40.7) | 68 470 (49.2) | 65 267 (39.6) | 25 165 (46.2) | 17 703 (46.0) | 32 478 (39.5) | |
| No information | 1 (0.0) | 5 (0.0) | 10 (0.0) | 11 (0.0) | 13 (0.0) | 11 (0.0) | 7 (0.0) | 59 (0.0) | |
| *Parity (n, %)* |  |  |  |  |  |  |  |  | |
| Nulliparous | 5 797(33.8) | 18 861 (31.8) | 44 650 (31.0) | 44 598 (32.1) | 58 376 (35.4) | 15 297 (28.1) | 9 036 (27.3) | 134 (32.9) | |
| Multiparous | 11 379 (66.2) | 40 469 (68.2) | 99 168 (69.0) | 94 509 (67.9) | 106 448 (64.6) | 39 104 (71.9) | 24 111 (72.7) | 273 (67.1) | |
| No information | 18 (0.1) | 44 (0.1) | 113 (0.1) | 87 (0.1) | 97 (0.1) | 29 (0.1) | 36 (0.1) | 65 (0.1) | |
| *BMI (n, %)* |  |  |  |  |  |  |  |  | |
| Underweight < 18.5 | 1 008 (4.0) | 1 868 (3.1) | 4 043 (2.8) | 5 019 (3.6) | 5 639 (3.4) | 2 434 (4.5) | 1 650 (4.3) | 2 963 (3.6) | |
| Normal weight (18.5–24.9) | 14 850 (58.7) | 38 021 (64.0) | 85 150 (59.2) | 78 762 (56.6) | 102 873 (62.4) | 29 316 (53.9) | 21 508 (55.9) | 48 462 (59.0) | |
| Overweight (25.0–29.9) | 5 384 (21.3) | 10 336 (17.4) | 29 774 (20.7) | 30 703 (22.1) | 32 802 (19.9) | 12 107 (22.2) | 7 934 (20.6) | 16 526 (20.1) | |
| Obese ≥ 30.0 | 3 056 (12.1) | 4 221 (7.1) | 15 409 (10.7) | 18 546 (13.3) | 17 523 (10.6) | 8 224 (15.1) | 5 087 (13.2) | 9 067 (11.0) | |
| No information | 993 (3.9) | 4 928 (8.3) | 9 555 (6.6) | 6 164 (4.4) | 6 084 (3.7) | 2 349 (4.3) | 2 284 (5.9) | 5 106 (6.2) | |
| *Smoking status (n, %)* |  |  |  |  |  |  |  |  | |
| Non-smoker | 20 289 (80.2) | 53 971 (90.9) | 122 673 (85.2) | 106 680 (76.6) | 142 198 (86.2) | 40 520 (74.4) | 28 992 (75.4) | 68 281 (83.1) | |
| Quit during the first trimester | 1 745 (6.9) | 1 895 (3.2) | 6 934 (4.8) | 9 578 (6.9) | 7 400 (4.5) | 3 238 (5.9) | 2 106 (5.5) | 4 018 (4.9) | |
| Smoker* | 2 595 (10.3) | 2 078 (3.5) | 11 010 (7.6) | 19 364 (13.9) | 11 500 (7.0) | 9 123 (16.8) | 63 20 (16.4) | 7 536 (9.2) | |
| No information | 662 (2.6) | 1 430 (2.4) | 3 314 (2.3) | 3 572 (2.6) | 3 823 (2.3) | 1 549 (2.8) | 1 045 (2.7) | 2 289 (2.8) | |

^a^ Row percentage. ^b^Column percentages. ^c^Data presented as number and percentage. All socioeconomic differences were statistically significant at P < 0.001. In socioeconomic status, Others included all unclassified occupations and socioeconomic status unknown, in smoking status, smoker refers to those who did not quit smoking during the first trimester*.*

Table S3: Multivariable logistic regression models of the association between maternal age and maternal obesity, stratified by socioeconomic groups

|  | <19 | 20-24 | 25-29 | 30-34 | >35 |
| --- | --- | --- | --- | --- | --- |
|  | OR | OR (95% CI) | OR (95% CI) | OR (95% CI) | OR (95% CI) |
| *Socioeconomic groups* |  |  |  |  |  |
| Upper-level employees | 1.00 (ref) | 1.71 (1.23–2.37) | 1.67 (1.21­–2.30) | 1.40 (1.02­–1.93) | 2.01 (1.46­–2.76) |
| Self-employed persons | 1.00 (ref) | 1.36 (1.10–1.69) | 1.42 (1.14–1.76) | 1.66 (1.32­–2.08) | 1.91 (1.53–2.38) |
| Lower-level employees | 1.00 (ref) | 1.79 (1.51­­–2.12) | 1.65 (1.39–1.95) | 1.66 (1.41–1.96) | 2.17 (1.84–2.56) |
| Manual workers | 1.00 (ref) | 1.88 (1.68–2.11) | 1.92 (1.72–2.15) | 2.11 (1.89–2.36) | 2.87 (2.57–3.21) |
| Students | 1.00 (ref) | 2.20 (1.54–3.15) | 2.34 (1.63­–3.34) | 2.37 (1.66–3.39) | 2.85 (1.99–4.07) |
| Long-term unemployed | 1.00 (ref) | 1.82 (1.53–2.17) | 2.02 (1.70–2.39) | 2.37 (2.00–2.82) | 2.86 (2.41–3.40) |
| Others | 1.00 (ref) | 1.73 (1.40–2.13) | 1.82 (1.49–2.22) | 2.04 (1.67–2.49) | 2.74 (2.23–3.36) |
| No information | 1.00 (ref) | 1.30 (1.09–1.57) | 1.42 (1.19–1.70) | 1.47 (1.23–1.76) | 1.87 (1.56–2.23) |

Women in the category of <19 years of age for each socioeconomic group was used as the reference category.


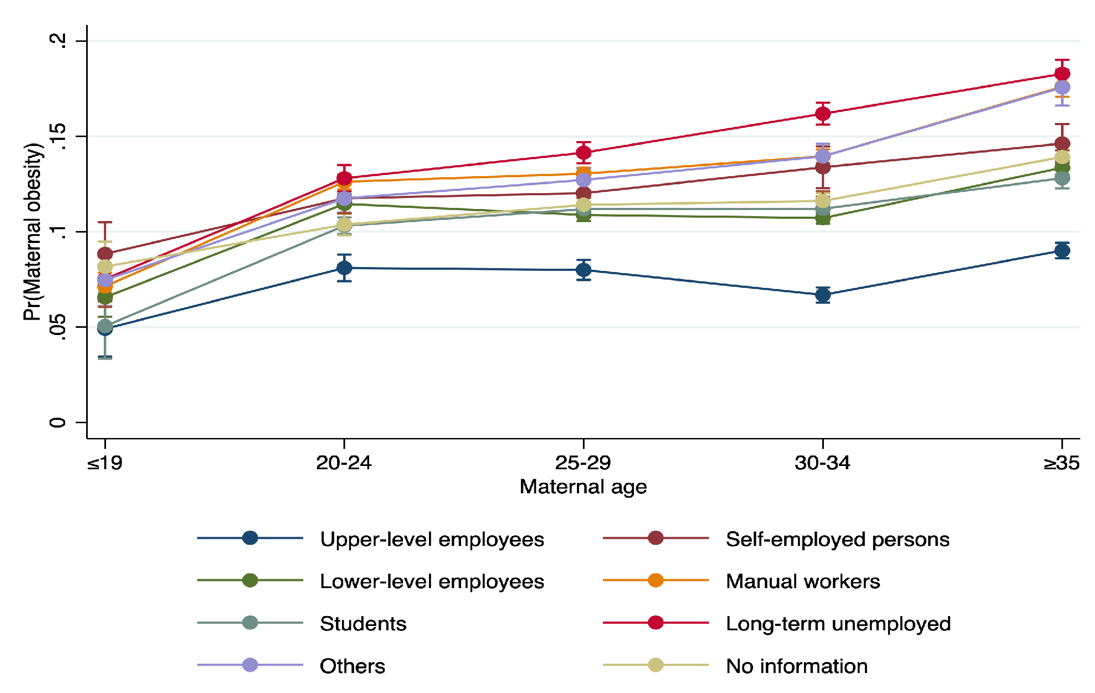


Figure S3 The probability of maternal obesity, predicted by age and socioeconomic groups. Women in the ‘upper-level’ category and maternal age <19 used as the reference groups. Adjusted for parity, smoking, and marital status. Error bars indicate 95% CIs.

Table S4: Three measures of interaction on an additive scale; Relative excess risk due to interaction (RERI), Attributable portion due to interaction (AP) and Synergy Index (SI) for maternal obesity

| Outcome  Maternal obesity | RERI | AP | SI |
| --- | --- | --- | --- |
|  | Estimates (95% CI) | Estimates (95% CI) | Estimates (95% CI) |
| Self-employed persons |  |  |  |
| 20-24 | 0.05 (-0.50–0.61) | 0.01 (-0.18–0.22) | 1.03 (0.74–1.43) |
| 25-29 | 0.08 (-0.45–0.63) | 0.03 (-0.16–0.22) | 1.05 (0.75–1.45) |
| 30-34 | 0.84 (0.30–1.38) | 0.25 (0.85–0.43) | 1.59 (0.03­­–2.48) |
| >35 | 0.72 (0.19–1.25) | 0.19 (0.04–0.35) | 1.37 (1.00­–1.87) |
| Low-level employees |  |  |  |
| 20-24 | 0.48 (0.13–0.83) | 0.18 (0.01–0.35) | 1.43 (0.91–2.25) |
| 25-29 | 0.35 (0.01–0.69) | 0.14 (-0.02–0.30) | 1.30 (0.87–1.96) |
| 30-34 | 0.59 (0.30–0.88) | 0.25 (0.08–0.42) | 1.76 (0.08­–0.42) |
| >35 | 0.79 (0.51–1.07) | 0.24 (0.12–1.37) | 1.56 (1.09–2.24) |
| Manual workers |  |  |  |
| 20-24 | 0.65 (0.37–0.92) | 0.22 (0.08–0.36) | 1.52 (1.03­–2.26) |
| 25-29 | 0.69 (0.46–0.93) | 0.24 (0.11–0.37) | 1.59 (1.06–2.39) |
| 30-34 | 1.19 (0.97–1.42) | 0.39 (0.27–0.52) | 2.47 (1.28–4.79) |
| >35 | 1.75 (1.39–2.11) | 0.42 (0.33–0.52) | 2.31 (1.55­–3.44) |
| Students |  |  |  |
| 20-24 | 0.50 (0.05– 0.95) | 0.21 (-0.006–0.44) | 1.61 (0.78–3.33) |
| 25-29 | 0.73 (0.33–1.13) | 0.29 (0.09–0.48) | 1.91 (0.90–4.07) |
| 30-34 | 1.14 (0.76­–1.5) \| | 0.41 (0.23–0.59) | 2.94 (0.98–8.74) |
| >35 | 1.03 (0.61­–1.45) | 0.31 (0.16–0.47) | 1.84 (1.12–3.00) |
| Long term unemployed |  |  |  |
| 20-24 | 0.60 (0.22–0.98) | 0.20 (0.04–0.36) | 1.45 (0.97–2.17) |
| 25-29 | 0.85 (0.52–1.18) | 0.27 (0.13–0.42) | 1.69 (1.09–2.63) |
| 30-34 | 1.70 (1.29–2.11) | 0.46 (0.35–0.58) | 2.83 (1.50–5.35) |
| >35 | 1.78 (1.32–2.24) | 0.42 (0.32–0.53) | 2.27 (1.51–3.42) |

Table S4 shows further analyses for the assessment of interaction on additive scale as explained by VanderWeele & Knol 2014.^1^ Women in the ‘upper-level’ category and maternal age <19 years used as the reference groups (ref) for maternal obesity in each four ‘exposure’ groups of women; for example, upper-level employees and manual occupations with women age <19 years and women in the category of 20 to 24 years (RERI = 0.65, AP = 0.22, SI = 1.52). The model was adjusted for variables parity, smoking, and marital status. If RERI and AP = 0 and SI = 1 there is no interaction on additive scale. These measures were used to assess whether the risk due to both exposures (maternal age and socioeconomic status) was greater than the sum of their individual risks. The RERI provides the direction of the effects and the AP and the SI provide information on the magnitude of the effects.

Table S5: Associations between maternal age and maternal obesity, test of reverse causality.

| Outcome |  |  |
| --- | --- | --- |
|  | OR  (95% CI) | Adjusted OR  (95% CI) |
| *Maternal age* |  |  |
| <19 | 1.00 (ref) | 1.00 (ref) |
| 20-24 | 1.66 (1.56–1.77) | 1.53 (1.43–1.63) |
| 25-29 | 1.67 (1.57–1.78) | 1.48 (1.39–1.58) |
| 30-34 | 1.73 (1.62–1.84) | 1.48 (1.39–1.58) |
| ≥35 | 2.18 (2.04–2.32) | 1.81 (1.70–1.93) |

Women who have received Medically Assisted Reproduction have been excluded from the analyses. Adjusted by parity. Abbreviations: OR Odds Ratio, CI confidence interval.

References:

1. VanderWeele TJ & Knol M J. A Tutorial on Interaction. Epidemiologic Methods 2014;3(1):33-72.
